# Cross-Validation of a New General Population Resting Metabolic Rate Prediction Equation Based on Body Composition

**DOI:** 10.3390/nu15040805

**Published:** 2023-02-04

**Authors:** Aviv Kfir, Yair Lahav, Yftach Gepner

**Affiliations:** Department of Epidemiology and Preventive Medicine, School of Public Health, Sackler Faculty of Medicine and Sylvan Adams Sports Institute, Tel-Aviv 6997801, Israel

**Keywords:** resting metabolic rate, prediction, equation, body composition

## Abstract

Current prediction equations for resting metabolic rate (RMR) were validated in a relatively small sample with high-individual variance. This study determined the accuracy of five common RMR equations and proposed a novel prediction equation, including body composition. A total of 3001 participants (41 ± 13 years; BMI 28.5 ± 5.5 kg/m^2^; 48% males) from nutrition clinics in Israel were measured by indirect calorimetry to assess RMR. Dual-energy X-ray absorptiometry were used to evaluate fat mass (FM) and free-fat mass (FFM). Accuracy and mean bias were compared between the measured RMR and the prediction equations. A random training set (75%, *n* = 2251) and a validation set (25%, *n* = 750) were used to develop a new prediction model. All the prediction equations underestimated RMR. The Cunningham equation obtained the largest mean deviation [−16.6%; 95% level of agreement (LOA) 1.9, −35.1], followed by the Owen (−15.4%; 95% LOA 4.2, −22.6), Mifflin–St. Jeor (−12.6; 95% LOA 5.8, −26.5), Harris–Benedict (−8.2; 95% LOA 11.1, −27.7), and the WHO/FAO/UAU (−2.1; 95% LOA 22.3, −26.5) equations. Our new proposed model includes sex, age, FM, and FFM and successfully predicted 73.5% of the explained variation, with a bias of 0.7% (95% LOA −18.6, 19.7). This study demonstrates a large discrepancy between the common prediction equations and measured RMR and suggests a new accurate equation that includes both FM and FFM.

## 1. Introduction

According to the World Health Organization (WHO), the global prevalence of overweight and obesity has nearly tripled over the last five decades, which is a significant public health concern [1]. Body weight reflects the balance between energy intake and energy expenditure. Assessment of resting metabolic rate (RMR), the main component (50–70%) of total daily energy expenditure (TDEE), is recommended to determine individual energy requirements [2]. Indirect calorimetry is the gold standard to measure RMR but is rarely used in clinical settings due to its high cost. Several prediction equations have been developed to predict RMR: Harris–Benedict [3], Food and Agricultural Organization/WHO/United National University [4], Mifflin–St. Jeor [5], and Owen et al. [6,7], have been developed as methods to assess RMR in clinical practice. Most of these equations estimate RMR based on sex and body weight, with the Harris–Benedict and Mifflin–St. Jeor equations also include height and age. Notably, body composition has a strong influence on energy expenditure [8], with a 4.5 kcal/day contribution per kg of fat, compared to a 13 kcal/day contribution (~×4) per kg of muscle [9]. Thus, using weight as a predictor, rather than body composition components, leads to a greater range of variance when estimating RMR and may provide a reasonable fit at the group level [10,11,12,13], but with a relatively large variance among individuals [13,14,15,16,17]. A significant issue is that while some studies report a trend to underestimation of RMR [10,12,16,18], there may also be problems with overestimation [14,17,19], depending on the characteristics of the study population. For instance, in one study conducted among 125 women [10], the equations resulted in an underestimate, although they yielded overestimates in another three studies with an African American population or Hispanic Women [14,17,19].

The Cunningham RMR prediction equation relies on fat-free mass (FFM) because of the strong correlation (r = 0.7) between FFM and RMR [20,21,22]. The equation was developed in 1980 and is based on the data of 223 subjects from various studies published by Harris and Benedict in 1919. The prediction ability of this method has been found to be better for athletes than for the general population [23] specifically among male as compared to female athletes (mean bias of 147 ± 283 kcal/day for males and 291 ± 333 kcal/day for females) [24]. To make the system more generally applicable, some studies have proposed models that integrate body composition measurements, but again these have primarily been based on small numbers of participants from specific populations [15,25,26].

Therefore, despite the wide use of the common equations, there remains a need to establish a novel prediction equation for RMR with a better fit at the individual level. The current study aimed to evaluate the common prediction equations on a large and diverse dataset and to develop a new prediction model that incorporates body composition parameters.

## 2. Materials and Methods

**Figure 1:**
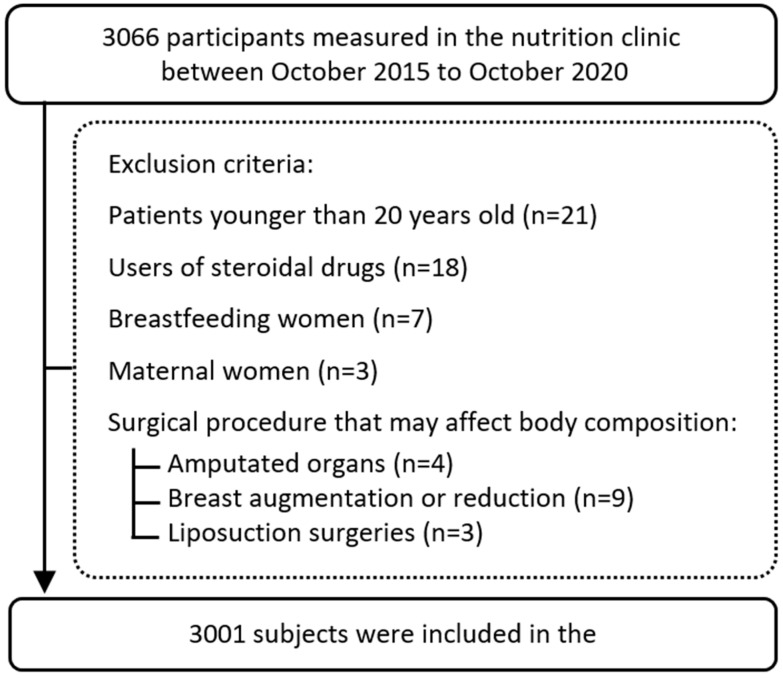
Study flow chart.

In this single-center cross-sectional observational study, 3001 participants attending a private nutrition clinic in the center of Israel were enrolled between October 2015 and October 2020. Study participants visited the clinic for nutrition consulting to improve their well-being, change lifestyle habits, or lose weight. Among the entire study population, 51.9% of the participants were male with a large range of BMI (14.7–59 kg/m^2^), and from different races, which represent well the general population in Israel. Participants were excluded from the study if they were lactating (*n* = 7), users of steroidal drugs (n = 18), or had undergone a surgical procedure that could affect body composition [e.g., amputated organs (*n* = 4), breast augmentation or reduction (*n* = 9), and liposuction surgeries (n = 3)]. Demographic parameters, including age and sex, were collected, as well as the medical history of bariatric surgery (*n* = 136), thyroid disorders (*n* = 46), or diabetes (*n* = 115) based on the clinical records. Furthermore, we classified the participants (*n* = 2937, 97.9% of the study sample) based on self-reported previous weight loss attempts: never (*n* = 2109), one or two attempts (*n* = 625), or three or more attempts (*n* = 203). The study flow chart is shown in Figure 1. Data collection and analysis were by the relevant ethical codes and were approved by the Ethics Committee of Tel Aviv University (0000607-2).

The same clinician conducted all the anthropometric measurements. While most participants (>95%) were tested on the same day, some conducted the metabolic and body composition assessments one week apart. Weight (±0.1 kg) was recorded on a digital scale (SECA mBCA 515; MFBIA; SECA^®^, Hamburg, Germany) while subjects were dressed in shorts and a T-shirt. A standard, wall-mounted stadiometer was used to measure the height (±0.1 cm) without shoes, and the BMI (kg/m^2^) was calculated accordingly. Neck and abdomen circumferences were measured (cm) with a flexible tape at the levels of laryngeal prominence and umbilicus, respectively.

Body composition measurements, FFM (kg), FM (kg), and FM (%), were measured using dual-energy X-ray absorptiometry (DXA, Lunar; GE) and analyzed using the integrated software (enCORE 2011, v.13.60.; GE, Madison, WI, USA). Participants were instructed to arrive at the clinic after at least a 4 h fast. Before the scan, participants were asked to remove all metal items. Each whole-body scan took ~7 min. Quality control calibration procedures were conducted on a spine phantom each morning.

RMR was measured in a metabolic cart using an indirect-calorimeter device, Quark RMR (Cosmed, Rome, Italy). Participants were instructed to arrive at the clinic by the morning after an overnight fast (12 h) and avoid any exercise training 24 h before the measurement. In addition, participants were restricted from consuming nicotine products for at least 2 h before the measurement. To ensure rest state when measuring RMR, according to the guidelines [27], participants were at rest 20 min before the measurement. Turbine calibration and gas calibration were performed before each test, according to the manufacturer’s instructions [28]. During the measurement, the subjects lay awake in a supine position, in a quiet room with stable temperature (22–24 °C). All measurements were conducted by adjusted size face mask. Measurements were 21 min long, with a 5 min adaptation phase excluded from the analysis, and the mean of the final 16 min calculated as the mean RMR. In case of major movement or falling asleep during the measurement, this period was excluded and the measurement continued to achieve the 16 min stability. Nearly all measurements (>90%) were at least 5 min steady state (10% or less coefficient of variation in VO_2_ and VCO_2_) [27]. VO_2_ and VCO_2_ were recorded every five second. The Weir equation was used to convert respiratory gas measures to energy expenditure, with acceptable respiratory exchange ratio ranged from 0.68 to 0.90 [27].

### Statistical Analysis

The normality of the distribution of each continuous variable was assessed using histograms and QQ plots and by the Kolmogorov–Smirnov Test. Variables found to have non-normal distributions were subjected to traditional transformations: square root for left-tail distributions and log-normal transformation for right-tail distributions. Participant characteristics were presented as the mean ± SD for continuous variables and by prevalence for categorical and dichotomic variables. Student’s *t*-test or Pearson’s Chi-squared test were used to comparing the sex differences. Bland–Altman analysis was used to determine the accuracy and the level of agreement of five common RMR equations (Harris–Benedict, Food and Agricultural Organization, WHO, United National University, Mifflin–St. Jeor, and Owen) with 95% level of agreement and mean bias [(RMR_measured_−RMR_estimated_/RMR_measured_) × 100] between the estimated RMR of each equation and the experimentally measured RMR from our data. Lin’s concordance correlation was used to examine the concordance between the r^2^ and the linear equation of each estimated equation and the experimentally measured RMR values. The level of inter-method agreement was compared using Bland–Altman plots, with a 95% level of agreement for mean bias.

To develop a multivariable prediction model for RMR, the dataset was randomly split using the train_test_split function of the scikit-learn Python package. A random subset of 75% of the participants (*n* = 2251) was assigned to a training set and 25% (*n* = 750) to a validation set. The two sets were matched in age, sex, BMI, and body composition (Supplementary Table S1). The inclusion variable for RMR predictors was set according to the Pearson’s coefficient between RMR and each variable and based on the least squares method to maximize r^2^. Parameters were included in the model according to a stepwise method, based on Pearson’s coefficient between RMR and each variable, and by the least squares method to maximize r^2^. Pearson’s correlation between potential variables was examined to avoid the exclusion of a multicollinearity variable with a high correlation (>0.7) in the model, and according to the variance inflation factor (VIF).

We validated our new model on the validation set (*n* = 750), using Bland–Altman plots (with 95% level of agreement for mean bias), and using Lin’s concordance correlation. A good model fit was defined as having maximum mean absolute error ±200 kcal/24 h (~10%). Data were collected using Microsoft^®^ Excel v.16.16.27 and analyzed using IBM^®^ SPSS Statistics v.27.

## 3. Results

Characteristics of the 3001 participants across sex are presented in Table 1. The mean age was 41 ± 13 years, between 20–95 years of age, with the following age distribution: 742 participants were between 20–30 years old, 712 were 30–40 years old, 806 participants were 40–49 years old, 478 participants were 50–59 years old, 200 participants were 60–69 years old, and 63 participants were 70 years old or older. The mean was BMI 28 ± 5.5 kg/m^2^ (range: 14.7–59 kg/m^2^), mean measured RMR was 1841 ± 365 kcal day^−1^, and 52% were females. As expected, compared to females, males had significantly higher RMR (2075 ± 325 kcal day^−1^ vs. 1615 ± 236 kcal day^−1^, *p* < 0.001) and FFM (64.1 ± 9.1 vs. 43.4 ± 6.4, *p* < 0.001), and lower FM (29.2 ± 9.3 vs. 39.5 ± 9.1, *p* < 0.001). The largest category of subjects was overweight (35.1%), followed by obesity (32.8%), normal weight (32.3%), and underweight (0.9%). The measured RMR, FFM, and FM increased significantly with increases in BMI (*p* < 0.001 for all).

**Figure 2:**
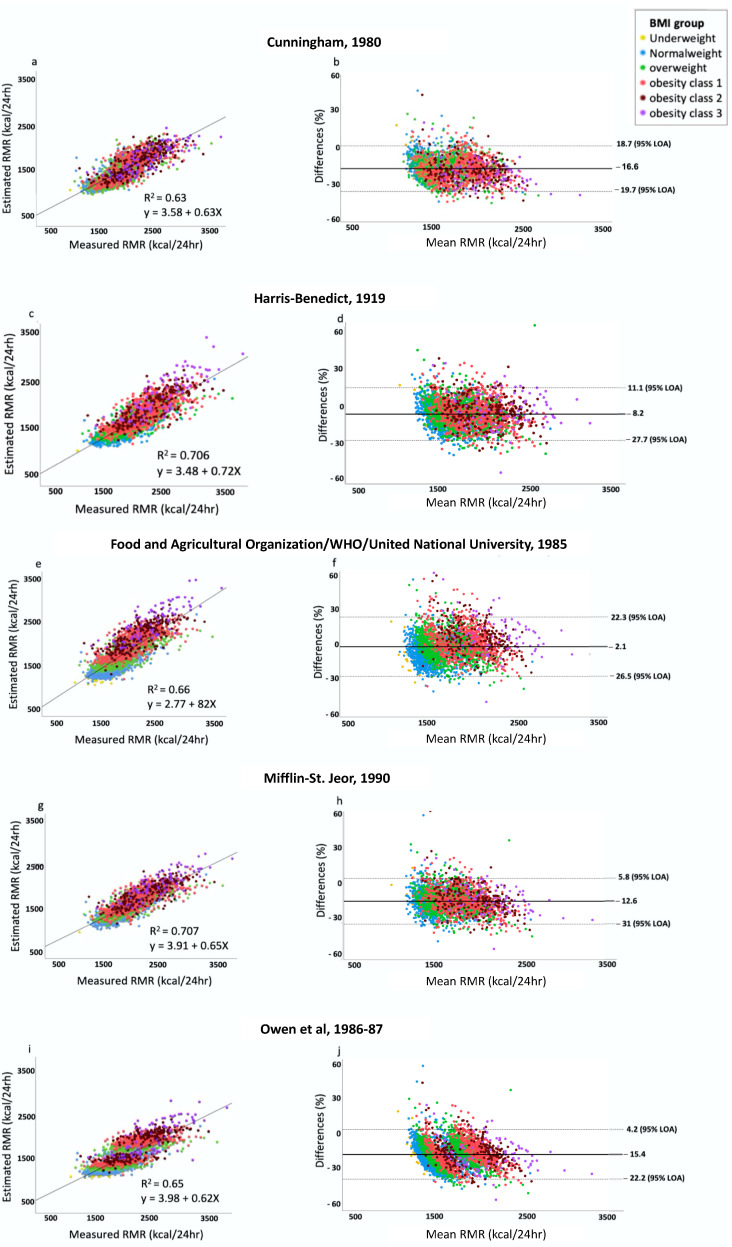
Bland–Altman analysis presenting the bias and the 95% level of agreement between each prediction equation and the measured RMR. The x-axis represents the mean of the measured and estimated RMR, and the y-axis expresses the difference in percentage between the RMR predicted using each of the equations and the RMR measured as follows: (pRMR-mRMR/mRMR)/100). Lin’s concordance correlation was used to determine the R^2^ and the linear equation between each common equation and the measured RMR. (**a**,**b**) Cunningham [20,22]; (**c**,**d**) Harris–Benedict [3]; (**e**,**f**) Food and Agricultural Organization/WHO/United National University [4]; (**g**,**h**) Mifflin–St. Jeor [5]; (**i**,**j**) Owen et al. equations [6,7].

Bland–Altman analysis presenting the bias and the 95% LOA of each of the common prediction equation is presented in Figure 2. The largest difference was obtained for the Cunningham equation, which is based on FFM as the single predicting factor (1521 ± 280 kcal day^−1^, −16.6%; 95% LOA 1.9, −35.1). This was followed by the Owen equation (1542 ± 281 kcal day^−1^, −15.4%; 95% LOA 4.2, −22.6), the Mifflin–St. Jeor equation (1593 ± 284 kcal day^−1^, −12.6; 95% LOA 5.8, −26.5), and the Harris–Benedict equation (1676 ± 313 kcal kg^−1^, −8.2%; 95% LOA 11.1, −27.7). The lowest mean deviation was obtained by the WHO/FAO/UAU equation (1792 ± 701 kcal kg^−1^, −2.1%; 95% LOA 22.3, −26.5). The explained variation (r^2^) ranges from 0.63 to 0.70, in the following order: Mifflin–St. Jeor > Harris–Benedict > WHO/FAO/UAU > Owen > Cunningham (Figure 2a,c,e,g). A high Pearson coefficient was found between RMR and weight (*p* = 0.74, *p* < 0.001), height (*p* = 0.65, *p* < 0.001), and neck circumference (*p* = 0.74, *p* < 0.001), with the highest coefficient obtained with FFM (*p* = 0.824, *p* < 0.001). In addition, a high Pearson coefficient was obtained between FFM and height (*p* = 0.8, *p* < 0.001), neck circumference (*p* = 0.86, *p* < 0.001), and weight (*p* = 0.65, *p* < 0.75). These were not combined as RMR predictors in the model in order to avoid multicollinearity.

The two newly proposed models are presented in Table 2. Model 1 includes age (years), sex, FFM (kg), and FM (kg) as predictors with an R^2^ value of 0.745.

For males, RMR (kcal/24 h) = 775.8 − (age × 5) + (FFM × 20.5) + (FM × 7.7)

For females, RMR (kcal/24 h) = 709 − (age × 5) + (FFM × 20.5) + (FM × 7.7)

Model 2 includes an interaction variable of FFM × FM, which increases the explained variation by 0.001.

For males, RMR (kcal/24 h) = 891.7 − (age × 5) + (FFM ×18.5) + (FM × 3.5) + (FFM × FM × 0.07)

For females, RMR (kcal/24 h) = 824 − (age × 5) + (FFM × 20.5) + (FM × 7.7) + (FFM × FM × 0.07)

In both models, the FFM predictor has the most significant contribution to RMR prediction (standardized β coefficients of 0.73 and 0.65, respectively; *p* < 0.001). Male sex was related to an increase of 9% in predicted RMR (*p* < 0.001); each year of age was related to a decrease of 5 kcal day^−1^, and 1 kg of FFM contributed 20.5 kcal day^−1^ to the RMR, which is a three-fold increase over the value for FM kg (7.7 kcal day^−1^, *p* < 0.001 for both). A sensitivity analysis for participants with thyroid disorders or following bariatric surgery revealed similar results in the accuracy of the model. Moreover, no significant change in the prediction accuracy or mean bias was found when the self-reported previous weight loss attempts were added to the model.

**Figure 3:**
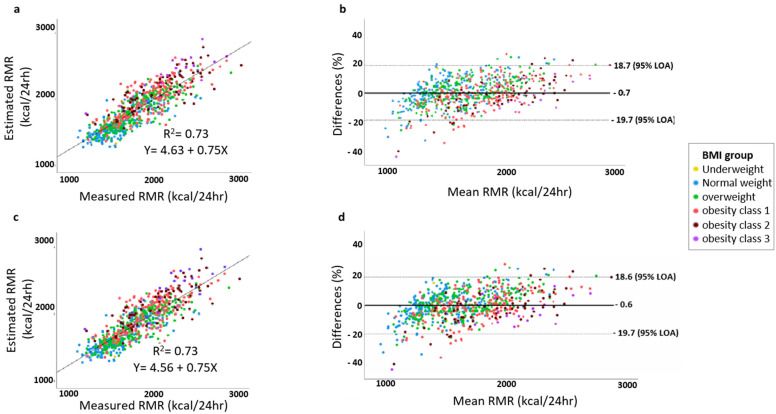
Bland–Altman analysis of the proposed new models applied to the validation set (*n* = 750). The x-axis represents the mean of the measured and estimated RMR, and the y-axis expresses the difference in percentage between the RMR predicted using each of the equations and the RMR measured as follows: (pRMR − mRMR/mRMR)/100). Lin’s concordance correlation was used to determine the R^2^ and the linear equation between each estimated equation and the measured RMR. (**a**,**b**) model 1, includes sex, age, FM, and FFM. (**c**,**d**) model 2, includes sex, age, FM, FFM, and the interaction between FM and FFM (FM*FFM).

Next, we applied Bland–Altman analysis to the validation set to determine the mean bias and level of agreement of both models (Figure 3). The new model (model 2) successfully predicted 73% of the explained variation of the measured RMR (*p* < 0.001). The mean deviation percentage of model 1 is −0.7% (*p* = 0.049), and no significant difference was found for the average deviation of model 2 and the measured RMR (average deviation −0.6%: 0.55, *p* = 0.123). The 95% level of agreement ranges from −18.6 to 19.7 for both proposed models.

## 4. Discussion

To our knowledge, the present investigation is the largest cross-sectional study to examine the accuracy of several common RMR prediction equations using body composition parameters, in addition to height, weight, sex, and age. As a result of the investigation, we propose a new prediction model for RMR. The known RMR prediction equations produced a large variation (−0.7% to −16.6%) in the mean bias with an explained variation of 0.63 to 0.70. In contrast, our new prediction equation, which includes both fat mass and fat-free mass, can successfully predict 73% of the explained variation of the measured RMR, with a mean bias of −0.7%.

Our findings demonstrate that the common prediction models underestimate experimentally measured RMR by −2.1 ± 12.4% to −16.1 ± 9.4%. While we appreciate that the population characteristics may influence the accuracy of a model, the characteristics of the 3001 Israeli participants comprising the study population employed are very similar to those of the general population. For example, the average BMI of the study participants was 28.5 kg/m^2^, similar to the mean BMI among the USA (28.5 kg/m^2^) [29] and slightly higher than the average BMI in Israel of (26.3 kg/m^2^). In addition, the sample size we used to determine the accuracy of the common equations and to develop the new prediction model is larger than that used in any previous study. The Harris–Benedict equation was developed based on 239 Caucasian participants with normal body weight [3]. Furthermore, these previous measurements were conducted under resting and not basal conditions, with no representation of elderly participants. Similarly, although the WHO/FAU/UNU equations were based on a large number of participants (2526), this population differed from the general population in that 90% of the participants were men and mostly young members of the military or police forces [4]. The Owen equation was based on 60 men and 44 women, with an age range from 18 to 82 years and 18 to 65 years, respectively, excluding more elderly women [6,7]. Similarly, the population used to develop the Mifflin–St. Jeor equation comprised 498 participants, with members of all the BMI categories, and ages 19–78 but did not include the oldest old group (>80). In addition, the development was based on data from the RENO Diet-Heart study, which involved a five-year follow-up. It might have introduced biases related to the induction process [5]. Accordingly, the proposed equation might be the most appropriate for the general population with a reasonable external validity.

The novel model proposed in this study exhibits higher accuracy with values of −0.6%; 0.55, and r^2^ of 0.73 compared to the 0.63–0.71 range of explained variation obtained from the commonly used equations. Notably, an extensive systematic review of validated common equations revealed large deviations between the predicted and measured values of RMR, with both under- and overestimations, depending on the equation and the study population [30]. The deviation generated by the Mifflin–St. Jeor ranged from an underestimate of 18% to an overestimate of 15% [31]; the deviation generated by the Harris–Benedict equation ranged from an underestimate of 65% to an overestimate of 43% among obese individuals [32]; and the Owen equation results ranged from 24% underestimation to 28% overestimation of the measured RMR [31]. Furthermore, the overestimation in predicting RMR tended to be particularly in people with obesity due to the higher fat mass, which has a lower metabolic rate. In this study, we did not find such an association. A possible explanation for this phenomenon may be the large age distribution in our study population, with the likelihood that some obese people were younger and had a high metabolic rate compared to older adults without obesity.

In this study, we found that combining demographic indices (age and sex) together with body composition indices increases the prediction accuracy and reduces the range of deviation. RMR prediction models based on body composition parameters have been previously proposed in the existing literature, but most are based on specific populations, e.g., women or athletes only [10,24]. The most common formula for use among the general population is the Cunningham equation, whose development was based on nine databases from nine different studies (a total of 1483 observations), where the data concerning body composition and energetic expense were collected using different methods in each study. When we examined the accuracy level and LOA, we found that of all the common equations examined, the Cunningham formula generated the most significant underestimate, 16.6% (95% LOA 35.1-, 1.9). This finding emphasizes that, despite the high correlation (r = 0.7) between FFM and RMR [20], combining the demographic indices, age, and sex with body composition parameters contributes to the accuracy of the individual RMR assessment and reduces the average deviation. Furthermore, we found that using body composition parameters (both FM and FFM) allows the equation to be applied to a wide range of populations, including individuals with obesity.

The model proposed in this study predicts 73% of the variation in RMR. Several factors influence RMR variation and may contribute to an increase in the explained variation. Several studies have shown that past calorie restriction attempts may lead to less-than-predicted RMR by losing muscle and respiring mass [33,34,35,36]. The metabolic effect of 5% weight loss remained even up to 6 years after the initial weight loss [37,38]. On the other hand, other studies did not find a long-term metabolic effect on weight reduction [39,40]. Although we hypothesized that past calorie restriction attempts would increase the explained variance, our models did not find an association between weight loss attempts and predicted RMR. Further and longitudinal studies should determine the role of the weight cycle on body metabolism and energy expenditure. Another unexplained variance in RMR may relate to our lack of ability to measure organ metabolic rate and size. The brain, liver, and kidneys have a relatively high mass-specific metabolic rate of ~240, 200, and 400 kcal/kg/day and account for about 22%, 21%, and 8% of RMR, respectively, in an average adult [41]. Moreover, studies in humans and mice found that weight loss or calorie restriction is associated with reduced internal organ size (except brain mass which increases in response to weight loss), which leads to metabolic adaptation [42,43]. Therefore, the high variability and residuals in RMR may be explained, at least partially, by differences in the size and the metabolic rate of internal highly metabolic organs.

The current study has several limitations. All the data were collected from a single clinic center in Israel, which might influence the ethnicity and socio-economic variety of the study Israeli population. However, our large sample size, with the representation of a wide age range, minimizes the potential selection bias in this study, as the study Israeli population has similar characteristics to the general population (same BMI and equal sex distribution). The strengths of this study include DXA measurements of body composition and the use of a pre-defined protocol with the same metabolic cart for the RMR measurements for all participants. The suggested new prediction equation in this study relies on DXA measurement to assess body composition, a gold standard but expensive assessment rarely used in a clinical setting. While BIA is correlated with DXA measurements of body composition [44,45], and might be used in the RMR equation derived in this study, this needs further testing. Accordingly, we suggest that the proposed model can be used based on measurements obtained from BIA devices. Unfortunately, data regarding the menstrual phase in women, which is well-known as a key factor in body metabolism and energy expenditure, were unavailable.

## 5. Conclusions

In summary, by combining body composition indices and demographic parameters, we present a novel model with higher individual accuracy for predicting RMR. Based on the study findings, we believe that the proposed prediction model represents an important tool that can be used for frequent measurements in the general population with access to body composition assessment.

## Figures and Tables

**Table 1:** Characteristics of the study population according to sex.

	Females *n* = 1559	Males *n* = 1442	*p*	Total *n* = 3001
Age (years)	40.3 ± 13.06	41.7 ± 13.01	*p* = 0.007	41 ± 13.01
Body weight (kg)	72.4 ± 15.4	91.1 ± 18.2	*p* < 0.001	81.3 ± 19.2
Height (m)	1.64 ± 0.06	1.77 ± 0.07	*p* < 0.001	1.7 ± 0.09
BMI (kg/m^2^)	27 ± 5.6	29 ± 5.3	*p* < 0.001	28.5 ± 5.5
FFM (kg)	43.4 ± 6.4	64.1 ± 9.1	*p* < 0.001	53.3 ± 13
FM (kg)	28.1 ± 11.7	26.5 ± 12.2	*p* < 0.001	27.3 ± 12
FM (%)	39.5 ± 9.1	29.2 ± 9.3	*p* < 0.001	34.5 ± 10.5
RMR (kcal/24 h)	1615 ± 236	2075 ± 325	*p* < 0.001	1841 ± 365
Abdominal circumference (cm)	96.1 ± 13.1	102 ± 14	*p* < 0.001	98.9 ± 13.9
Neck circumference (cm)	32.9 ± 2.6	40 ± 3.5	*p* < 0.001	36.5 ± 4.7
Diabetes	37 (2.4%)	78 (5.4%)	*p* < 0.001	115 (3.8%)
Hypothyroidism	109 (7%)	27 (1.9%)	*p* < 0.001	136 (4.5%)
Bariatric surgery, yes (%)	26 (1.7%)	20 (1.4%)	*p* = 0.53	46 (1.5%)
BMI Group *				
Underweight	20 (1.3%)	8 (0.6%)	*p* = 0.038	28 (0.9%)
Normal weight	642 (41%)	326 (22.6%)	*p* < 0.001	968 (32.3%)
Overweight	507 (32.5%)	546 (37%)	*p* = 0.002	1053 (35.1%)
Obesity class 1	247 (15.8%)	385 (26.7)	*p* < 0.001	632 (21.1%)
Obesity class 2	101 (6.5%)	129 (8.9%)	*p* = 0.011	230 (7.7%)
Obesity class 3	41 (2.6%)	48 (3.3%)	*p* = 0.26	89 (3%)

Continuous variables presented as mean ± SD, and as prevalence for categorical and dichotomic variables. Independent samples, Student’s *t*-test, or Person’s Chi-squared test were used to compare the results in females and males. * underweight; BMI < 18.5 (kg/m^2^), normal weight; BMI 18.5–24.9 (kg/m^2^), overweight; BMI 25–29.9 (kg/m^2^), obesity class 1; BMI 30–34.9 (kg/m^2^), obesity class 2; BMI 35–39.9 (kg/m^2^); obesity class 3; BMI > 40 (kg/m^2^). FFM, fat-free mass; FM, fat mass; RMR, resting metabolic rate.

**Table 2:** This table presents two new models developed to predict RMR, based on the training set (*n* = 2251, 75%).

	Model 1			Model 2		
	Unstandardized β	Standardized Coefficient β	*p*	Unstandardized β	Standardized Coefficient β	*p*
Constant	709	-	*p* < 0.001	824	-	*p* < 0.001
Age (years)	−5	−0.18	*p* < 0.001	−5	−0.18	*p* < 0.001
sex (male)	66.8	0.09	*p* < 0.001	67.7	0.09	*p* < 0.001
FFM (kg)	20.5	0.73	*p* < 0.001	18.5	0.65	*p* < 0.001
FM (kg)	7.7	0.25	*p* < 0.001	3.5	0.11	*p* = 0.016
FFM (kg) × FM (kg)	-	-	*p* < 0.001	0.07	0.16	*p* = 0.003
R^2^	0.745			0.746		

Adjusted *R^2^* was 74.5 % for model 1, and 74.6% for model 2 calculated by linear regression based on stepwise elimination. FFM, fat-free mass; FM, fat mass; RMR, resting metabolic rate.

## Data Availability

According to the code of ethics, the data described in the manuscript, the code book, and the analytic code will not be available, out of respect for the patients’ personal information.

## Supplementary Materials

The following supporting information can be downloaded at: https://www.mdpi.com/article/10.3390/nu15040805/s1, Table S1: Study characteristics between the training set and validation set.

## Abbreviations

BEE	basal energy expenditure
BIA	bioelectrical impedance analysis
BMI	body mass index
DXA	dual-energy X-ray absorptiometry
FAO	Food and Agricultural Organization
RMR	resting metabolic rate
T2DM	type 2 diabetes
TDEE	total daily energy expenditure
TEE	total energy expenditure
UNU	United National University
VIF	variance inflation factor
WHO	World Health Organization
