# Supplementary material for: Cross-Validation of a New General Population Resting Metabolic Rate Prediction Equation Based on Body Composition
Source: Nutrients. 2023 Feb 4;15(4):805. doi: 10.3390/nu15040805 (PMC9960966; doi:10.3390/nu15040805)
Supplement: Supplementary file 1 [file nutrients-15-00805-s001.zip › nutrients-2170786-supplementary.pdf]

nutrients

Supplementary Materials

Cross-validation of a New General Population Resting Metabolic Rate Prediction Equation Based on Body Composition

Aviv Kfir, Yair Lahav and Yftach Gepner *

Table S1. Study characteristics between the training set and validation set.

	Training set N = 2251	Validation set N = 750	*p*-value*	Total N = 3001
Age (years)	41 ± 13	40.9 ± 13	$p = 0.502$	41 ± 13
Sex, males (n, %)	1088 (48.3)	354 (47.2)	$p = 0.61$	1442 (48.1)
Body weight (kg)	81.1 ± 19.2	82.2 ± 19.4	$p = 0.876$	81.3 ± 19.2
Height (m)	1.7 ± 0.1	1.7 ± 0.1	$p = 0.36$	1.7 ± 0.1
BMI (kg/m^2)	28 ± 5.5	28.2 ± 5.6	$p = 0.31$	28.5 ± 5.5
FFM (kg)	53.2 ± 13	53.8 ± 12.9	$p = 0.72$	53.3 ± 13
FM (kg)	27.2 ± 11.9	27.7 ± 12.1	$p = 0.36$	27.3 ± 12
FM (%)	34.5 ± 10.5	34.6 ± 10.6	$p = 0.89$	34.5 ± 10.5
RMR (kcal/24h)	1836 ± 368	1854 ± 65	$p < 0.001$	1841 ± 365

Continuous variables percentage as mean ± SD, and prevalence for categorical and dichotomic variables.

*For Training set vs. validation set comparison we use independent samples, Student's t-test or Person’s Chi-squared test respectively.

BMI, body mass index; FFM, fat-free mass; FM, fat mass; RMR, resting metabolic rate.
